# Impact on the reduction of CO2 emissions due to the use of telemedicine

**DOI:** 10.1038/s41598-022-16864-2

**Published:** 2022-07-22

**Authors:** César Morcillo Serra, Ana Aroca Tanarro, Catherine Mary Cummings, Araceli Jimenez Fuertes, José Francisco Tomás Martínez

**Affiliations:** 1 Medical Direction, Sanitas Digital Hospital, Ribera del Loira, 52, Madrid, Spain; 2 Corporate Affairs Department, Sanitas & Bupa Europe and Latam, Ribera del Loira, 52, Madrid, Spain

**Keywords:** Climate sciences, Health care

## Abstract

Digital health can reduce CO_2_ emissions thanks to telemedicine and access to digital test results and medical reports. However, the environmental impact of digital health activity is not well known. Here, we show that telemedicine reduces CO_2_ emissions. We found a net total of 6,655 tons of CO_2_ emissions decrease through a reduction in patient travel to surgeries and medical clinics thanks to the alternatives of digital appointments and digital access to test results and medical reports, which avoid the need to travel to a clinic for a face-to-face visit or to pick up printed results or reports. During 2020, a total of 640,122 digital appointments were carried out by the health care company, which avoided 1,957 net tons of CO_2_ emissions, while patients downloaded 3,064,646 digital medical reports through the company portal, which avoided an additional 4,698 net tons of CO_2_ emissions. Our results demonstrate how digital appointments and digital reports, reduce CO_2_ emissions by reducing the need for patient travel.

## Introduction

Digital health is the use of information and telecommunication technologies to improve health. It is a large umbrella that incorporates a broad range of developments, such as telemedicine, mobile health and other solutions, including online appointments and access to digitalised medical reports. Digital health enables innovative models of care and allows health care professionals to put patients at the centre of medical assistance, improving efficacy and accessibility^1^. Digital tools have the potential to simplify both administrative and health care processes, to improve the quality and reduce the cost of medical care and to break down geographical barriers. Several international organisations recommend further developing and implementing digital health strategies^2^.

Digital health has been our ally during the COVID-19 crisis^3^. There has been a shift from an almost exclusively analogical attention to an effective—and accepted—level of digital attention, through virtual appointments (essentially video calls carried out by a doctor through a secure platform that is also connected with a patient’s medical history record and the ability to prescribe follow-up tests, scans and pharmaceutical prescriptions)^4^.

One of the most developed areas of digital health is telemedicine. It can be defined as remote medical assistance and implies the use of information and communication technologies to boost communication both between doctors and patients and among health professionals themselves^5^.

Digital consultation is the best-known example of telemedicine; it comprises teleconsultation (audio communication) and video consultation (audio and video communication). Digital consultation improves the patient experience and promotes the patient’s empowerment with regard to his or her own care^6^. Satisfaction levels are very high^7^. It also avoids carbon dioxide (CO_2_) emissions in those cases when it avoids the need to travel to a surgery for a face-to-face appointment, and although the digital process does lead to an increase in energy consumption, this is far outweighed by the benefits of the avoided travel.

CO_2_ is one of the most important greenhouse gases; it traps heat in the atmosphere and is one of the main gases responsible for climate change. The impact of climate change is evident in every continent and poses a risk both for human health and for nature^8^. Human activities have been responsible for almost all the increase in greenhouse gases in the atmosphere in recent years. The transportation sector generates the largest share of greenhouse gas emissions, which come mainly from the burning of fossil fuels for cars, trucks, ships, trains and airplanes. Electricity production generates the second largest share of greenhouse gas emissions. Many mitigation strategies have been proposed that centre primarily on the reduction of fossil fuels and energy demand^9^. Although transportation for health reasons only represents 1.5% of the total number of trips ^10^, digital health could help address both issues, especially telemedicine programs, due to the potential to avoid patient travel^11,12^.

In the current climate of growing interest in reducing the environmental footprint of health care activities^13^, this study evaluates the environmental impact of digital health solutions, analysing the net CO_2_ emissions avoided thanks to the corresponding reduction in patient travel to medical clinics when using a digital consultation or when downloading medical reports instead of travelling in to request printed versions.

## Results

**Figure 1:**
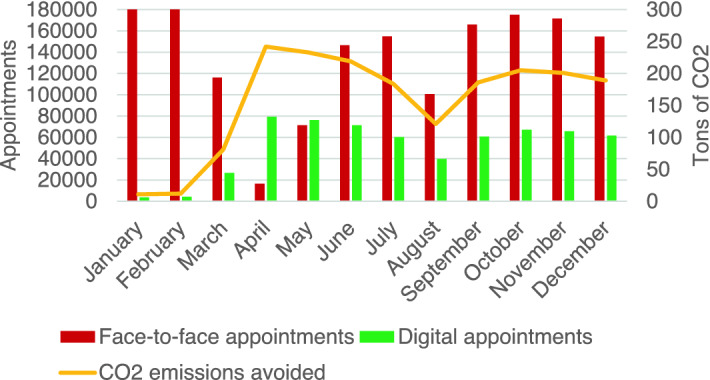
Number of appointments and CO_2_ emissions avoided. Evolution of the number of face-to-face and digital appointments made monthly during 2020. The yellow line shows the monthly CO_2_ emissions avoided, resulting from multiplying the number of digital appointments by the average savings of 3.057 kg of CO_2_ for every digital appointment.

The total number of medical appointments carried out by the health care company with patients in 2020 was 3,015,530. Of those, 640,122 were in a digital format (495,913 were video appointments and 144,209 were telephone appointments), with an average of 3,700 digital appointments per day. This digital use was estimated to have avoided 1,957 net tons of CO_2_ emissions (Fig. 1). Similarly, in 2020, patients downloaded 3,064,646 medical reports to their digital health folder, with an estimated impact of 4,698 net tons of CO_2_ emissions avoided. These two digital health solutions together avoided 6,655 net tons of CO_2_ emissions in 2020.

Of the digital appointments, 66,510 were with a generalist practitioner (GP), and 573,612 were with a specialist. A total of 74.7% of the video appointments were made from a mobile phone application, and 25.3% were made over the web using a computer.

The average age of patients who requested a digital appointment was 39 years old, unlike those who used a face-to-face consultation, which was 44 years old. The people who most used the digital appointment option during 2020 were people between 30 and 39 years old (54,812 digital appointments), followed by those between 45 and 54 (47,372 digital appointments). It is worth noting, however, that, overall, this service was used by patients of all age groups, with those over 70 years old making more than 18,000 video appointments during 2020.

Patient satisfaction with the use of digital appointments, measured using the net promoter score (NPS) methodology, was high, with an average rating of 62.1% in 2020 and 48.7% of customers repeating this communication method.

## Discussion

This study showed that the 640,122 digital appointments carried out and the 3,064,646 digital medical reports downloaded by the customers in 2020 avoided an estimated 6,655 net tons of CO_2_ emissions, according to the calculations revised by the Carbon Trust. These results confirm that digital health reduces the impact of health care on the environment by reducing the number of car journeys for a face-to-face visit or to pick up paper medical reports, contributing to both population and planet health.

Our results are consistent with those from Vidal-Alaball et al.^11^ involving 9,034 teleconsultations with a reduction of 29,384 net tons of CO_2_—representing an average of 3.248 kg saved per journey, in line with our estimate of 3.057 kg in Spain. Other studies also suggest that the reduction in visits as a result of telemedicine services and the consequent reduction of pollutant emissions are significant^14,15^.

Digital consultations have risen exponentially since 2020 to enable continuity of care during the COVID-19 pandemic. During this test period, it proved to be a safe and effective alternative to classical care^7^. Telemedicine, beyond being considered a very useful means in exceptional situations, provides advantages that will lead us to rethink its use in day-to-day care processes outside of the exceptionality of a pandemic. These advantages benefit both the patient and the professional and the health care system. In 2020, users highlighted its ease of use, flexibility, comfort and savings in time and money by reducing travel, with the consequent environmental impact achieved by avoiding CO_2_ emissions, in addition to greater security by reducing the risk of contagion throughout the different COVID-19 waves or other infectious diseases. These advantages were also experienced by health care personnel, who reported greater work–life balance thanks to remote working and considered digital tools a well-accepted health care alternative^7^. Most users who made virtual appointments consider that they offer quality medical care^4^, affirm that they intend to continue with this type of care once the pandemic is over^16^ and believe that its implementation will be permanent^17^. Telemedicine also brings benefits to health care organisations by moving care from health care facilities to homes and mobile devices. It increases the efficiency of the consultations because they are shorter once the rapid learning process to use the new software has been overcome. It improves accessibility to services by ensuring continuity of care, reducing waiting lists and prioritising the patients who need it most, directing them towards adequate health care circuits and providing the right attention to the right patient^7^. At the same time, it allows agile communication among health care levels, avoiding unnecessary referrals^18^. In addition to the advantages perceived by the different users, there is evidence that telemedicine improves the health of the population^19^ and reduces the costs derived from health care, especially in certain chronic diseases such as diabetes^20^ or cardiovascular diseases^21^.

The high satisfaction of users and the high recurrence of the use of digital consultation in our sample coincide with those reported in the scientific literature^4^. These results favour the maintenance of this level of activity once the situation has normalised. Thus, we have seen the consolidation of a new way of communication between patients and doctors that ensures equality of access to health care while reducing in-person hospital visits and their environmental impact.

The effect of reduced travel and its decrease in CO_2_ emissions has been clearly demonstrated during the pandemic. COVID-19 has meant a digital transformation in health, but it has also had an impact on human activities and, in turn, energy use and CO_2_ emissions. There was an abrupt 8.8% decrease in global CO_2_ emissions (~ 1,551 MT) in the first half of 2020 compared to the same period in 2019. However, the pandemic’s effects on global emissions diminished as lockdown restrictions relaxed and some economic activities restarted^22^.

The Spanish public National Health System covers 45,207,196 inhabitants of Spain. Every year, it takes care of approximately 234 million GP consultations and 103 million specialised medical consultations^23^. Telemedicine alone cannot solve all clinical demands, but according to some studies, up to 75% of face-to-face visits can be resolved remotely^24^. A profound change in the health care model will be necessary to reach this figure; however, if we take the digital consultation rate of 25% witnessed in our sample and extrapolate this rate to apply it to telehealth solutions across Spain, we can guess that it could have led to 84.2 million digital consultations had it been used throughout the health system. With each digital consultation avoiding 3.057 kg of net CO_2_ emissions, by avoiding the need for patients to travel to an appointment, this would have meant avoiding 78,717 net tons of CO_2_ emissions in Spain. This means 0.03% of the annual production of 252.6 million tons in the country^25^. Even though this figure might seem modest, with increased take-up and by extending it to an increasing number of examples, digital health has the potential to be considered a mitigation strategy for climate change^26^. Every little step helps towards becoming a climate-neutral country and having a healthier planet.

Approximately 2.5 million cases of noncommunicable diseases attributable to air pollution are predicted by 2035 if atmospheric pollutants remain at their current levels, making air pollution an important public health priority^27^. The concept of One Health recognises that the health of people is closely connected to the health of animals and our shared environment. To take care of people's health, we must also take care of the health of the planet. Therefore, the health sector must also assume the responsibility of caring for people, animals and environmental health if it truly wishes to achieve a healthy society.

The results of this study should be interpreted with caution and in the context of its limitations. The calculation of the net CO_2_ emissions data has been obtained from the different modes of travel used by the population in Spain and the UK; therefore, these results cannot be extrapolated to other countries with other uses of the means of transport. For the purposes of this study, the type of transport used has been taken to be a normal vehicle with average emissions. The use of hybrid or electric cars, or higher emissions through the use of outdated and obsolete cars, has not been taken into account. The calculation of distance travelled and the use of different means of transport is an average based on the average distance travelled by all health care company patients in 2019. It is not based on actual distances or methods used. Air pollution is a mixture of different pollutants. CO_2_ is clearly the most important, and we selected it as the only marker of air pollution. Methane is the second greatest contributor to anthropogenic climate change, but the contribution of methane emissions from vehicles to climate change is 0.3% of that of CO_2_ emissions^28^. Although the benefits of telemedicine have been discussed, there are other aspects that can have an influence, such as a reliable telecommunications system or that a face-to-face visit is more suitable for the diagnosis of certain conditions than videoconferences, and therefore,digital consultations do not always avoid a face-to-face appointment. But in 90% of cases in our sample, no face-to-face visit occurred in relation to the same type of specialist in the week following the telemedicine consultation, leading us to conclude that the digital consultation successfully resolved the patient request. For the analysis, it has been assumed that having digital reports means that they are not printed, but some patients could still print them at home. Another limitation is the population bias. This report is based on a relatively small population in Spain covered by one insurance plan, but it almost certainly applies in other places with a telemedicine health care system. Moreover, Sanitas policyholders are concentrated in the main cities of the country, so the observed impact of digital health on CO_2_ savings cannot be generalised to rural areas, where it would be higher due to the greater distances that users must travel to go to health centres and where the effectiveness of telemedicine is even higher^11^.

This study confirms that digital health, specifically digital consultation and the download of digital vs. paper reports, reduces the environmental impact of health care by reducing the number of journeys for a face-to-face visit and the number of printed medical reports. Further studies should include more information on other sustainable modes of transport and include air pollution in the cost-effectiveness analysis of face-to-face visits compared to telemedicine.

Nowhere are the effects of climate change manifesting more clearly than in human health. The modern health care sector contributes to this serious phenomenon and, at the same time, is being affected by it. Ameliorating the health care sector’s environmental effects and reducing greenhouse gas emissions could not only improve health for everyone but also reduce the costs of care^29^.

The present study was thus conducted to identify one of the multiple ways in which the health sector can contribute to preventing climate change. The expansion of telemedicine programs should be considered an option as part of a global strategy to reduce the emission of atmospheric pollutants. As everything indicates that telemedicine is here to stay, digital consultation will be a key tool to solve not only the health challenges of the future but also the environmental challenges.

## Methods

This is a retrospective study analysing the environmental impact of the digital health activity of Sanitas insurance policyholders in Spain in 2020. Sanitas is a private Spanish health care company with approximately 2 million policyholders and a hospital network with 29 health care facilities and teaching hospitals, with 3,136 doctors from 35 specialties available. Sanitas professionals have clinical protocols for the use of digital consultation by specialty and offer the health system 24 h a day, 7 days a week.

Sanitas policyholders have access to different digital health solutions through a mobile application and a website; they are able to schedule a doctor’s appointment, download their medical test results or reports and access the digital consultation platform. Video consultation is accessible using smartphones or computers, while teleconsultation is feasible using any kind of phone.

To calculate the net CO_2_ emissions data avoided thanks to the patient’s switch to digital processes, we worked with the Carbon Trust, a global climate change and sustainability consultancy. We reviewed the statistical data of the average distances travelled by Sanitas customers to get from their homes to a doctor’s surgery, and with these data, they calculated the average distance covered nationally (approximately 13 km for a round trip). The methodology included a review of the different modes of travel used by the population, according to Spain’s and UK’s national statistics registers^30,31^. Different means of transport were analysed (walking, cycling, bus, train, car) with the following considerations. In the large provinces (Madrid, Barcelona and Valencia), 50% of the clients would go by car, 21% by bus, 26% by train or metro and 3% by a nonpolluting method of transport (foot, bicycle). In the rest of the provinces, 80% would go by car, 9% by bus, 8.0% by train and 3% by a nonpolluting method of transport.

The Carbon Trust’s methodology included in its equation the CO_2_ emissions generated by using digital tools for video appointments (energy consumption of equipment and data transmission) or to download and save a digital medical report. The emissions related to videoconferencing were subtracted from the total avoided emissions to establish a “net” quota of avoided CO_2_.

To calculate the emissions generated during a video consultation, the energy consumption of a videoconference was estimated to last an average of 9.5 min and was calculated according to the energy intensity of the data centres, data transmission and the use of devices, in line with the reports of the International Energy Agenda. The emission factor of the Spanish electricity mix was used.

The savings derived from the use of paper were calculated based on 2 aspects. (1) Use of paper: emissions related to unused paper were calculated by multiplying the average weight of paper saved in each report by the production emission factor of paper. Emissions at the end of the useful life of the paper have also been taken into account, multiplying the Spanish statistics of paper and cardboard waste on recycling, landfills and incineration by the related emission factors and the weight of the paper. (2) Energy for printing: The energy saved by avoiding the printing of paper in each consultation was calculated by multiplying the average consumption of electricity to print an A4 page by the average number of sheets of medical reports. Emissions avoided by printing were obtained by multiplying the electricity emission factor by the energy saved by printing.

**Figure 2:**
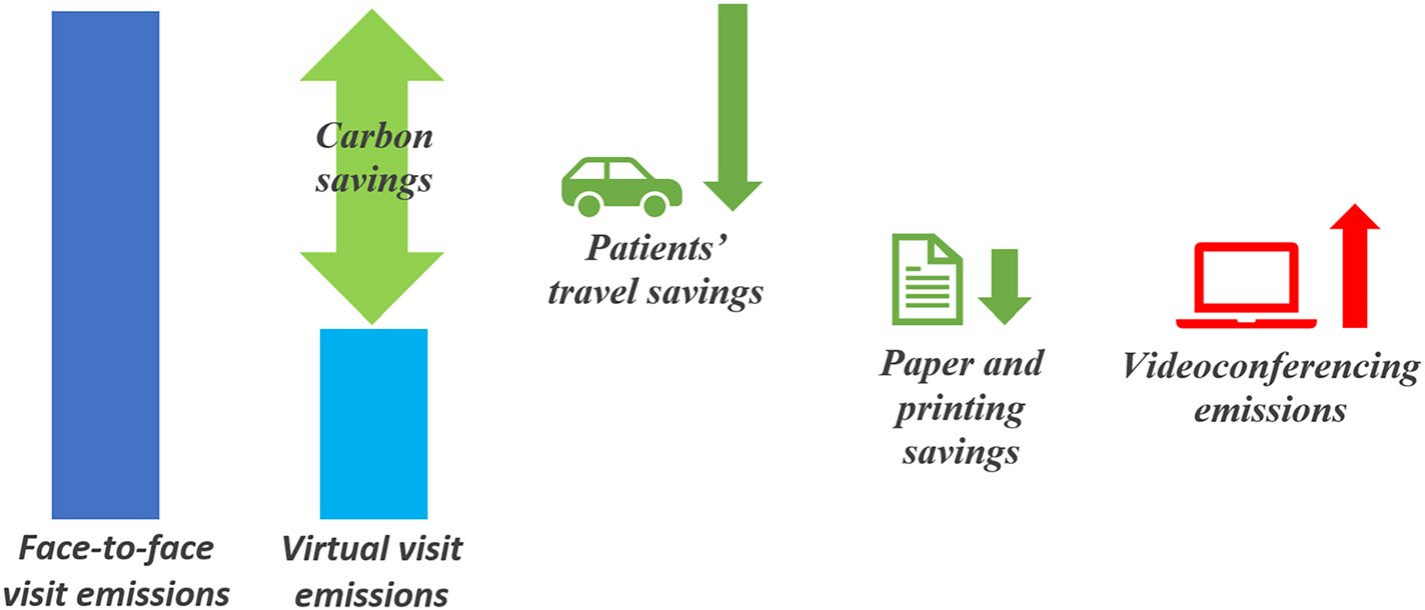
Calculation of carbon savings of performing a visit online. The net carbon savings of performing a visit online were calculated by summing the carbon savings derived from the avoided patient travel and document printing and subtracting the videoconferencing emissions.

The net carbon savings for digital appointments and downloaded reports were calculated by summing the carbon savings derived from the avoided patient travel and document printing and subtracting the videoconferencing and downloading emissions, as shown in Fig. 2. Performing a digital consultation reduced carbon emissions primarily (> 99%) through reduced patient travel. A small fraction (< 1%) of carbon savings was also derived from the avoidance of using paper for printing the visit’s results.

The result of the calculation described above gave an average of 3.057 kg of net CO_2_ emissions avoided for every digital appointment and 1.5 kg avoided for every medical report downloaded instead of being physically collected in the clinic. After each video appointment, patients can access information on the CO_2_ they have avoided if their video appointment has avoided their need to use a car to travel in for a face-to-face appointment. The message to users, in the personal area of the digital platform, reads: “If this visit has saved you a trip to the practice, you have avoided 3.1 kg in CO_2_ emissions”.

Microsoft Excel and Microsoft Power BI were used for data processing and quantitative analysis.

### Ethics declarations

There was no human participation or human data involved in the study.

## Data Availability

The datasets generated and/or analysed during the current study are not publicly available because they include private data about the activity of the insurance company but are available from the corresponding author on reasonable request.
